# High-power-efficiency and ultra-long-lifetime white OLEDs empowered by robust blue multi-resonance TADF emitters

**DOI:** 10.1038/s41377-025-01750-z

**Published:** 2025-02-11

**Authors:** Guohao Chen, Jingsheng Miao, Xingyu Huang, Zhenghao Zhang, Zhuixing Xue, Manli Huang, Nengquan Li, Xiaosong Cao, Yang Zou, Chuluo Yang

**Affiliations:** Shenzhen Key Laboratory of New Information Display and Storage Materials, College of Materials Science and Engineering, Shenzhen University, Shenzhen, 518060 China

**Keywords:** Organic LEDs, Polymers

## Abstract

White organic light-emitting diodes (WOLEDs) show very promising as next-generation light-sources, but achieving high power efficiency (PE) and long operational lifetime remains challenging because of the lack of stable blue emitters that can harvest all triplet (T_1_) excitons for light emission. Herein, we propose integrating stable azure multi-resonance thermally activated delayed fluorescent (MR-TADF) emitters into tri-color hybrid WOLEDs to tackle these issues. By meticulously selecting MR-TADF emitters and precisely tuning the exciton recombination zone, the optimized tri-color devices based on BCzBN-3B achieve color-stable white light emission with maximum external quantum efficiency (EQE_max_) and maximum PE (PE_max_) of 34.4% and 101.8 lm W^−1^, respectively. Furthermore, the LT_90_, defined as the time for the luminance to drop to 90% of its initial value at 1000 cd m^−2^, reaches 761 h. In addition, a hybrid WOLED with deep blue emitter developed using our strategy achieves a high color rendering index of 88 and an EQE_max_ of 30.6%, further demonstrating the versatility and effectiveness of our approach. The record-breaking efficiency and ultra-long lifetime underscore the success of hybrid white-light devices by incorporating robust blue MR-TADF emitters. These advancements open new avenues for commercialization of hybrid WOLEDs, presenting promising solutions for energy-efficient lighting and display technologies.

## Abstract (web summary)

A tri-color emitting layer design incorporating robust blue multi-resonance emitters creates high-performance white light-emitting diodes with EQE_max_ of 34.4%, PE_max_ of 101.8 lm W^−^^1^, and LT_90_ value of 761 h.

## Graphical abstract

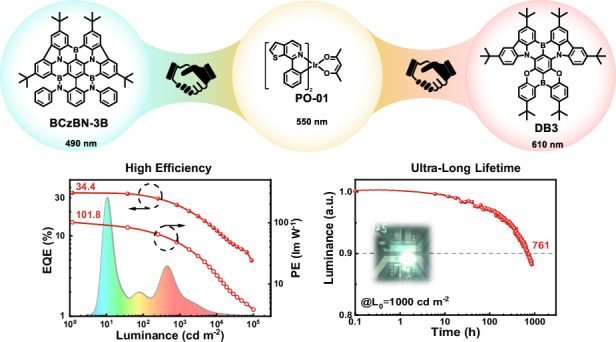

## Introduction

White organic light-emitting diodes (WOLEDs) have been regarded as highly promising next-generation lighting sources by virtue of their attractive merits including low power consumption, high flexibility, and eye-protection^1–3^. Over the past decade, significant advances have been achieved for WOLEDs in terms of their electroluminescence efficiency with state-of-the-art external quantum efficiencies (EQEs) over 30%, by using phosphorescent emitters that can harvest both electrically generated singlet (S_1_) and triplet (T_1_) excitons for light emission^4–6^. However, the lack of stable blue phosphors significantly limits the large-scale commercialization of phosphorescent WOLEDs, despite the great advances of green to red phosphorescent emitters^7–10^. To advance the real-world applications of WOLEDs, long device lifetime is of utmost importance^11^. Nonetheless, at this stage, the inadequate operational lifetime of WOLEDs remains a formidable obstacle to their industrialization, which is closely related to both the device configuration and the inherent chemical stability of the emitters, particularly the blue ones^12,13^. From the perspective of energy transfer and carrier transport process, a device structure that could ensure efficient energy cascade, broad recombination zone, as well as balanced carrier transport should be ensured. To this end, many strategies have been explored, such as utilizing exciplex systems, p-i-n junctions, tandem structure, down-conversion, and exciton-confining structures, etc^14–20^.

In terms of chemical stability of emitters, the blue phosphors are the bottleneck of phosphorescent WOLEDs, which results from the inherent structural instability of blue phosphorescent emitters with high triplet energy levels^21,22^. Fortunately, hybrid WOLEDs containing blue fluorescence emitters could offer a promising solution, in which the blue emitters could be conventional fluorescence, triplet-triplet annihilation (TTA), and thermally activated delayed fluorescence (TADF) emitters^23–25^. For example, it could be a feasible strategy to use conventional blue fluorescent (CBF) emitters with longer operational lifetimes, combined with stable green and red phosphorescent emitters. However, since blue CBF emitters could only harness singlet excitons which account for 25% of the electrically generated excitons as dictated by spin statistics, resulting in low internal quantum efficiencies (IQEs) and power efficiencies (PEs)^26^. Additionally, the low-lying triplet states of CBFs lead to unstable emissions under various driving conditions, necessitating the design of more sophisticated device structures or strategies to address this issue^27^. For blue TTA emitters, the theoretical maximum IQE is only 62.5%, which also leads to mitigated device efficiencies^28^. Interestingly, blue-emitting exciplex hosts through an intermolecular donor-acceptor structure could also be an alternative^29,30^, which is expected to provide balanced carrier transport and a broader exciton recombination zone (RZ), thus a prolonged device lifetime. However, controlling the exciton recombination zone for high device stability remains a formidable challenge, and blue exciplexes with high electroluminescence (EL) efficiencies and stable structures are rare. Based on the above discussion, stable blue TADF emitters which can harness both S_1_ and T_1_ excitons to achieve 100% IQE could be a viable option^31–34^. Importantly, the blue TADF emitters could be purely organic compounds. On the one hand, the purely organic structures enable the blue TADF emitters to be more chemically stable, as compared to their phosphorescent counterparts which typically suffer from relatively weak metal-ligand coordination bonds^35^. On the other hand, the purely organic structures are more economical, which is beneficial to reduce the fabrication costs of WOLEDs. Specifically, blue multi-resonance TADF (MR-TADF) emitters, which feature rigid ring-fused aromatic frameworks, exhibit both high structural stability and high EL efficiency, and these emitters are highly promising in achieving better device lifetimes^36,37^. This suggests that blue MR-TADF emitters may be preferred for the next generation stable WOLEDs^38^.

**Fig. 1:**
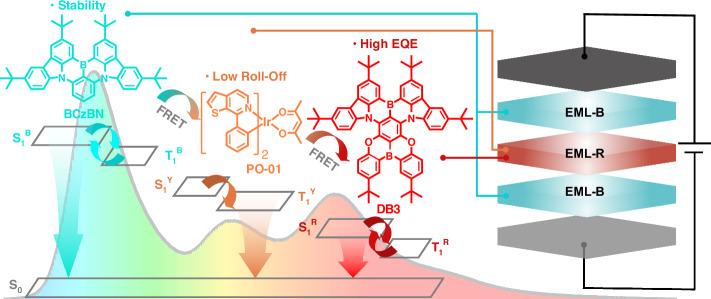
Schematic illustrations of device strategy for sandwich structure B-R-B WOLED. WOLEDs are fabricated with robust MR-TADF emitters as blue emitters in EML-B, and PO-01 as yellow sensitizer for red emitter DB3 in EML-R. Efficient Förster energy transfer (FRET) among emitters results in high efficiencies and stable emission spectra

Herein, hybrid WOLEDs based on blue MR-TADF emitters with high efficiencies and excellent operational lifetimes were fabricated. We selected a highly efficient sky-blue MR-TADF emitter of BCzBN with good structural stability due to its large and rigid π-conjugated core skeleton, as the blue component in the emitting layer (EML-B)^39^. A red MR-TADF emitter DB3 with a para-B-π-B configured skeleton was chosen as the red emitter, as recently reported by our group. Devices based on DB3, with the assistance of a sensitizer, exhibited excellent EL performance and an extraordinarily long operational lifetime^40^. In this work, DB3 in the red emitting layer (EML-R) was equipped with the same yellow phosphorescent sensitizer iridium (III) bis(4-phenylthieno[3,2-c]pyridinato-N,C2’)acetylacetonate (PO-01), exhibiting a dual-color emission at an optimal concentration pairing by using sensitization strategy. Meanwhile, we proposed a sandwich EML structure Blue-Red-Blue (B-R-B) to address the variation of emission spectra with driving conditions in devices (see Fig. 1). BCzBN-based devices demonstrated an outstanding maximum external quantum efficiency (EQE_max_) and maximum power efficiency (PE_max_) of 32.7% and 92.0 lm W^−1^, respectively. Notably, BCzBN was subsequently replaced with the sky-blue MR-TADF emitter BCzBN-3B^41^, which has a higher horizontal orientation factor, photoluminescence quantum yield (*Φ*_PL_) and faster reverse intersystem crossing rate (*k*_RISC_). The optimized hybrid WOLED with BCzBN-3B devices achieved a superior 34.4% of EQE_max_, 101.8 lm W^−1^ of PE_max_ and Commission Internationale de l'Éclairage (CIE) 1931 coordinates of (0.31, 0.46). Based on the same strategy, optimized lifetime-test devices still maintained a high level of EQE, while featuring remarkable device stability and operational lifetime as well. The results indicate that MR-TADF materials have significant potential for application in the field of WOLEDs, opening new avenues for material selection and offering new device structure strategies for future breakthrough in commercialized WOLEDs.

## Results

Since blue MR-TADF emitters play a key role in our hybrid WOLEDs, it is important to evaluate their EL performance before using them to fabricate hybrid WOLEDs. Monochromatic OLED based on the selected blue MR-TADF emitter of BCzBN was firstly fabricated with the structure of ITO/HAT-CN (5 nm)/1,1-bis[(di-4-tolylamino)phenyl]cyclohexane (TAPC, 30 nm)/tris(4-carbazolyl-9-ylphenyl)amine (TCTA, 15 nm)/3,3-bis(N-carbazolyl)-1,1’-biphenyl (mCBP, 10 nm)/2 wt% BCzBN: 5-(3-(4,6-diphenyl-1,3,5-triazin-2-yl)phenyl)-7,7-dimethyl-5,7-dihydroindeno[2,1-b]carbazole (DMIC-TRZ) (45 nm)/tris(benzene-3,1-diyl)tris(diphenylphosphine oxide) (PO-T2T, 20 nm)/ 1-(4-(10-([1,1′-biphenyl]-4-yl)anthracen-9-yl)phenyl)-2-ethyl-1H-benzo[d]imidazole (ANT-BIZ, 30 nm)/Liq (2 nm)/Al (100 nm). The molecular structures of the materials used were depicted in Fig. S1. Both TAPC and TCTA were applied as hole-transport layers. ANT-BIZ served as the electron-transport layer. mCBP and PO-T2T functioned as exciton-blocking layers. DMIC-TRZ was selected as the host material in EML due to its balance hole-electron transporting property, which was beneficial for achieving a unity carrier balance ratio. As a result, the optimized device (denoted as P1) based on BCzBN displayed sky-blue emission peaking at 490 nm with a full width at half maximum (FWHM) of 28 nm, corresponding to CIE coordinates of (0.09, 0.41), as illustrated in Fig. S2 (Supporting Information). Device P1 achieved a high EQE_max_ value of 33.1%. To test the stability of BCzBN in device, the operational lifetime of the BCzBN-based device was measured at an initial luminance of 1000 cd m^−2^ (device configuration seen in Fig. S3, Supporting Information). It was found that the LT_80_ (the duration required for the luminance to decrease to 80% of the initial value) was 746 h, which substantiated our confidence in accomplishing stable hybrid WOLEDs.

**Fig. 2:**
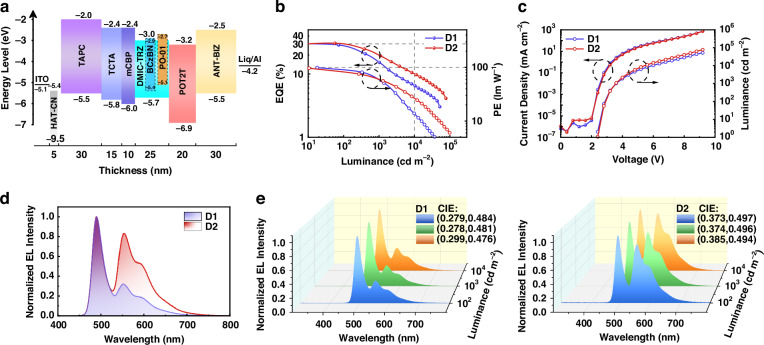
Device data and EL performance of dual-color WOLEDs based on BCzBN. **a** Device architecture and energy diagram. **b** EQE-luminance and PE-luminance (EQE-L-PE) curves of devices D1(0.3 wt% PO-01: 5 wt% BCzBN) and D2(0.5 wt% PO-01: 5 wt% BCzBN). **c** Current density-voltage and luminance-voltage (J-V-L) curves of devices D1 and D2. **d** EL spectra of devices D1 and D2 measured at a luminance of 1000 cd m^−2^. **e** EL spectra and CIE (x, y) coordinates of devices D1 and D2 measured at a luminance of 100, 1000 and 10,000 cd m^−2^

Inspired by the excellent blue EL performance of BCzBN as demonstrated above, it was then employed in hybrid WOLEDs. Firstly, dual-color WOLEDs using BCzBN as the blue component and PO-01 as the orange component were fabricated following the optimized device configuration depicted in Fig. 2a: ITO/HAT-CN (5 nm)/ TAPC (30 nm)/ TCTA (15 nm)/ mCBP (10 nm)/EML (25 nm)/PO-T2T (20 nm)/ANT-BIZ (30 nm)/Liq (2 nm)/Al (100 nm). Particularly, the thickness of EML was decreased to 25 nm to derive a low driving voltage and thus a high PE. Specifically, the EML consisted of DMIC-TRZ (host), BCzBN (blue component), and PO-01 (orange component). Device D1 contained 0.3 wt% PO-01 and 5 wt% BCzBN in DMIC-TRZ, while the concentration of PO-01 was increased to 0.5 wt% in device D2. The device performances are shown in Fig. 2. As shown in Fig. 2b and c, both devices achieved low turn-on voltage of 2.4 V and high efficiencies, with EQE_max_ of 30.3%/30.6% and PE_max_ of 99.0/91.2 lm W^−1^ for device D1/D2. D1 and D2 exhibited typical two-component spectral profiles with distinct emission originating from BCzBN and PO-01 (Fig. 2d), demonstrating the effective energy cascade in the EML. The CIE coordinates at 1000 cd m^−2^ were (0.28, 0.48) and (0.37, 0.50) for D1 and D2, respectively. Interestingly, the emission spectra and CIE coordinates of both devices remained remarkably stable under various driving conditions corresponding to different luminance levels (Fig. 2e), indicating the efficient energy transfer among DMIC-TRZ, BCzBN, and PO-01. The high efficiencies and stable spectra of these devices validate the combination of the EML system comprising DMIC-TRZ, BCzBN, and PO-01. In addition, it was clearly demonstrated in Figs. 2d and e that increasing the doping ratio of PO-01 from 0.3 wt% to 0.5 wt% could dramatically enhance the color rendering index (CRI) of the devices, with CRI values of 35 and 46 for device D1 and D2, respectively. However, it should be noted that the CRI values achieved by the two devices were moderate resulting from the deficiency of the red spectral component, which is the typical case for dual-color WOLEDs based on complementary-color strategy^26^.

To compensate for the deficiency of the red component in the spectrum and thus increase the CRI values, we introduced the red MR emitter of DB3 into EML to fabricate the tri-color WOLEDs. DB3 was selected due to its suitable red emission with EL peak at 606 nm, excellent EL performance and ultra-long operational lifetime when paired with PO-01 (sensitizer) and DMIC-TRZ (host), as reported recently by our group. To explore the energy transfer from PO-01 to DB3, we prepared three thin films: Film I with only PO-01 (1 wt% PO-01: DMIC-TRZ), Film II with both PO-01 and DB3 (0.3 wt% DB3: 1 wt% PO-01: DMIC-TRZ) and Film III with only DB3 (0.3 wt% DB3: DMIC-TRZ). We investigated the energy transfer using transient photoluminescence (PL) measurements. As shown in Fig. S13 and Table S4 (Supporting Information), Film II exhibited a shorter exciton lifetime (988 ns) compared to Film I (1158 ns) at the emission wavelength of 550 nm, indicating efficient energy transfer from PO-01 to DB3. The FRET rate constant (*k*_FRET_) for Film II was calculated to be 1.49 × 10^5^ s^−1^, which was lower than the radiative decay rate (*k*_r_) of PO-01, which is 7.51 × 10^5^ s^−1^. This result suggested that PO-01 could sensitize DB3, enhancing radiative exciton generation in DB3 while transferring energy to DB3 without completely quenching the emission of PO-01. As a result, the PO-01 emitter continued to emit light, generating a broad spectrum that spans both yellow and red wavelengths. The broad emission spectrum including yellow and red light was advantageous for coupling with blue light, facilitating the creation of white light emission.

**Fig. 3:**
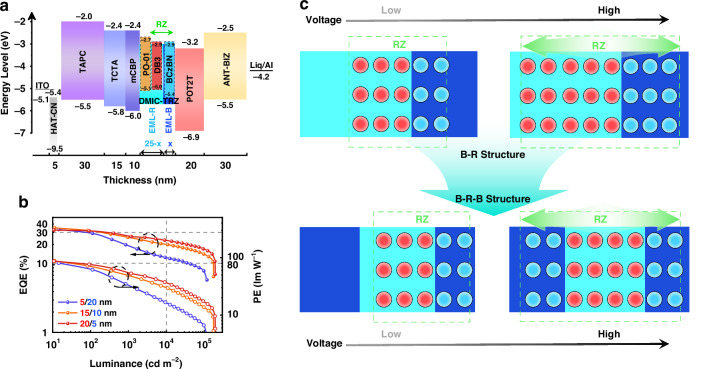
Device architecture, EL performance and the exciton recombination behaviors of tri-color WOLEDs. **a** Device architecture and energy diagram of tri-color devices. **b** EQE-luminance and PE-luminance (EQE-L-PE) curves of devices contained EML-R/B with different thickness. **c** Schematic diagram of the exciton recombination zone at low and high voltage in tri-color devices with B-R and B-R-B structure

Accordingly, 0.3 wt% DB3: 1 wt% PO-01: DMIC-TRZ was selected as the red EML (EML-R) of the tri-color devices after concentration screening, while 10 wt% BCzBN: DMIC-TRZ as the blue EML (EML-B) (Fig. 3a). With the total EML thickness fixed at 25 nm, devices with EML-B thickness of 15, 10, and 5 nm were fabricated, and the EQE-L and PE-L of the devices are shown in Fig. 3b. It turned out that the devices all achieved white light with EQE_max_ values over 30%, despite their different EL spectral profiles. The emission spectra of all devices fluctuated considerably at different luminance levels, with a high proportion of blue emission at low brightness and a dominance of red component at high brightness. As shown in Fig. S4 (Supporting Information), when the EML-B thickness was set to 15 nm, the red emission was barely observed at a low luminance of 100 cd m^−2^. This indicated that the exciton RZ was biased towards the EML-B side. A white-light CIE coordinate of (0.32, 0.46) and an enhanced CRI value of 48 were achieved at 1000 cd m^−2^. As the EML-B thickness was reduced, the red emission gradually increased at low luminance levels. When reduced to 10 nm, devices exhibited CIE coordinates of (0.36, 0.46) and a boosted CRI value of 58. With the EML-B thickness of 5 nm, the red component was tuned to have a considerable portion out of the overall spectrum, suggesting that the exciton RZ was located near the interface between EML-R and EML-B. The higher CRI of 65 was obtained by these devices with typical warm-light CIE coordinates of (0.45, 0.46). However, despite the improved CRI, a significant drawback of these devices was that all of them showcased extremely unstable emission spectra. The net effect of the increased driving voltage was a broadened RZ, where a large number of excitons were captured in the EML-R leading to a surge in the red component (Fig. 3c). This phenomenon was proven to depend on the device structure rather than the emitters (Fig. S5, Supporting Information). Therefore, the spectral stability of the devices could be addressed through the structural design. A practical strategy is to divide the EML-R into two layers to form a B-R-B structure, in which the high-energy blue emitting layers at both ends could confine the excitons to the red layer with a low-lying energy level. This configuration would make it easier to obtain a stable emission spectrum in the constrained RZ.

**Fig. 4:**
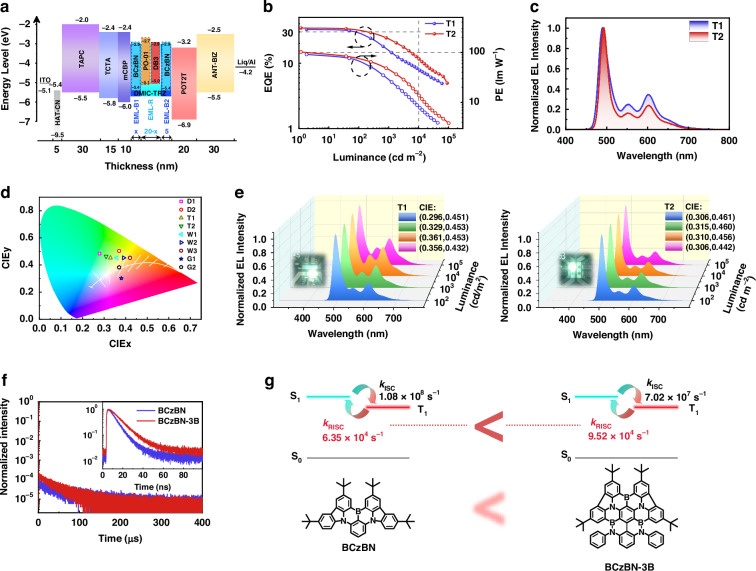
Device data and EL performance of tri-color WOLEDs with B-R-B structure. **a** Device architecture and energy diagram of devices with B-R-B structure. **b** EQE-luminance and PE-luminance (EQE-L-PE) curves of devices T1 and T2. **c** EL spectra of devices T1 and T2 measured at a luminance of 1000 cd m^−2^. **d** Summary of CIE coordinates for all hybrid WOLEDs. **e** EL spectra for each of devices T1 and T2 measured at a luminance of 100, 1000, 10,000 and 100,000 cd m^−2^. The insets show the photograph of each device at 10^3^ cd m^−2^. **f** Transient photoluminescence decay curves (300 K) of 2 wt% emitters in DMIC-TRZ film under inert atmosphere. **g** The calculated *k*_RISC_ and *k*_ISC_ rate for BCzBN and BCzBN-3B

Aiming to improve the spectral stability, a tri-color EML structure with a B-R-B configuration was then proposed (Fig. 4a). This structure comprises three layers: EML-B1 (10 wt% BCzBN: DMIC-TRZ) adjacent to the hole transport layer, EML-B2 (10 wt% BCzBN: DMIC-TRZ) adjacent to the electron transport layer, and EML-R (0.3 wt% DB3: 1 wt% PO-01: DMIC-TRZ) sandwiched between the above two blue layers. The remaining layers were kept intact for the devices. Such an EML design not only aimed to limit the expansion of RZ under high driving voltages, but also to minimize the emission contribution from EML-R and complement the blue emission when the RZ widened. Based on previous results of RZ modulation, the thickness of EML-B2 was fixed at 5 nm, and the total thickness of EML-B1 and EML-R was set to 20 nm. As a result, the red component of the spectrum was significantly suppressed at high brightness with the incorporation of EML-B1 (Fig. S6, Supporting Information). When the thickness of EML-B1 was 15 nm, the red and blue portions of the spectrum were balanced at different brightness levels and thus the stable spectra at high driving voltages, which benefited from the additional exciton recombination in the EML-B1 region, as elucidated in Fig. 3d. With the EML-B1/R/B2 thickness of 15/5/5 nm, the device (denoted as T1) demonstrated quite stable EL spectra with increasing brightness, as was the case for the CIE coordinates (Fig. 4e). At the brightness of 1000 cd m^−2^, device T1 exhibited the CIE coordinates of (0.33, 0.45) featuring a white light with a CRI value of 52, demonstrated in Figs. 4c and d. However, device T1 suffered from large efficiency roll-off, dropping from EQE_max_ of 32.3% to only 14.4% at the brightness of 1000 cd m^−2^, which was probably due to the relatively long delayed fluorescence lifetime (*τ*_d_) of BCzBN which dominated in the emission. Decreasing the doping concentration of BCzBN was unable to address the problem of severe efficiency roll-off, depicted in Fig. S7 (Supporting Information).

Based on the above discussion, it was expected that replacing BCzBN with another blue MR-TADF emitter with a shorter *τ*_d_ would alleviate the roll-off of device efficiency. Thus, the blue MR-TADF emitter of BCzBN-3B was selected^41^. We prepared the doped films for BCzBN-3B and BCzBN in DMIC-TRZ and compared their photophysical properties. It was found that BCzBN-3B exhibited a higher *Φ*_PL_, a shorter *τ*_d_ of 40 μs compared to 76 μs from BCzBN, and thus a faster *k*_RISC._ The detailed photophysical properties were provided in Figs. 4f and g and Table S2 (Supporting Information). The monochromatic OLED based on BCzBN-3B exhibited sky-blue EL peaking at 493 nm with an excellent EQE_max_ of 41.1%, which was superior to that of BCzBN (Fig. S8, Supporting Information). In addition, the device maintained an EQE of 29.5% at 1000 cd m^−2^ corresponding to a roll-off of only 28%, compared with the value (56%) of the BCzBN-based device. More importantly, BCzBN-3B achieved an LT_80_ of 858 hours at an initial luminance of 1000 cd m^−2^, which was longer than that of BCzBN-based device. These results clearly suggested the superior EL performance and better stability of BCzBN-3B than BCzBN. Therefore, BCzBN was replaced by BCzBN-3B as the blue emitter for further investigation. The optimal concentration of BCzBN-3B was explored in Fig. S9 (Supporting Information), indicating that the effect of concentration on the emission spectrum was negligible. The optimized tri-color device based on 10 wt% BCzBN-3B with EML structure of B-R-B was denoted as T2. As depicted in Fig. 4b, device T2 achieved an outstanding EQE_max_ of 34.4%, which to the best of our knowledge represented the highest EQE ever reported for WOLEDs without using light outcoupling structure to date (Table S3, Supporting Information). The higher EQE_max_ of device T2 compared to device T1 could be attributed to the superior properties of the BCzBN-3B blue emitter. Specifically, BCzBN-3B had a higher horizontal orientation factor of 94%, *Φ*_PL_ of 95% and a faster *k*_RISC_, since the only difference between T1 and T2 was the blue emitter. The higher horizontal orientation factor of BCzBN-3B enhanced light out-coupling efficiency, while the increased *Φ*_PL_ and faster *k*_RISC_ both contributed to a higher internal quantum efficiency. Statistical data on EQE_max_ and PE_max_ for T1 and T2 were provided in Fig. S14 (Supporting Information). In addition, device T2 presented better suppression of efficiency roll-off compared to device T1, attributable to the faster *k*_RISC_ of BCzBN-3B relative to BCzBN. Device T2 produced a cold white emission with CIE coordinates of (0.31, 0.46), a correlated color temperature (CCT) of 5953 K and a CRI value of 44, measured at a luminance of 1000 cd m^−2^ (Fig. 4d). To evaluate the flexibility of such an EML structure, three devices were fabricated with varying EML-R thicknesses: 10 nm (W1), 15 nm (W2), and 20 nm (W3). These devices were produced by increasing the EML-R thickness while maintaining the other layers unchanged based on the structure of device T2, and they successfully achieved highly efficient white light emission, with EQE_max_ values over 30% and suppressed roll-offs (Fig. S10, Supporting Information), demonstrating the superiority of the device structure for hybrid WOLEDs. The higher V_on_ values for devices W1, W2 and W3, compared to D1, D2, T1 and T2, are mainly due to the thicker EML-R and trap states from low-bandgap PO-01 and DB3 in the wide-bandgap host DMIC-TRZ. Almost constant emission spectra from 100 to 100,000 cd m^−2^ and higher CRIs were also obtained in warm white device configurations. The key EL data of all hybrid WOLEDs in this work are summarized in Table 1, and the device configurations are listed in Table S1 (Supporting Information).

**Table 1:** Summary of hybrid WOLEDs EL data

Device	V_on_^a^ [V]	EQE_max/1000_^b^ [%]	CE_max/1000_^b^ [cd A^−1^]	PE_max/1000_^b^ [lm W^−1^]	CIE^c^ [x, y]	CCT^d^ [K]	CRI^d^	L_max_^e^ [cd m^−2^]
D1	2.4	30.3/15.2	76.7/38.1	99.0/33.2	(0.28, 0.48)/(0.30, 0.48)	6864	35	51,964
D2	2.4	30.8/19.4	87.0/54.3	101.8/47.4	(0.37, 0.50)/(0.38, 0.49)	4695	46	77,306
T1	2.4	32.3/14.4	68.1/33.0	89.1/25.9	(0.33, 0.45)/(0.36, 0.45)	5615	52	63,777
T2	2.4	34.4/23.4	77.8/46.6	101.8/44.8	(0.31, 0.46)/(0.31, 0.45)	5953	44	90,137
W1	2.6	33.4/24.1	71.5/54.1	80.2/42.5	(0.36, 0.45)/(0.36, 0.45)	4887	49	129,271
W2	2.6	32.4/25.3	70.5/57.7	79.1/45.4	(0.39, 0.45)/(0.39, 0.45)	4204	55	139,999
W3	2.6	31.7/25.7	69.1/59.1	77.5/46.4	(0.42, 0.45)/(0.41, 0.45)	3607	61	159,943
G1	2.7	32.1/14.6	64.0/29.8	74.5/26.0	(0.38, 0.30)/(0.37, 0.29)	3120	71	64,558
G2	2.7	30.6/16.0	75.8/38.2	88.2/33.4	(0.37, 0.38)/(0.37, 0.36)	4196	88	57,807

^a^Turn-on voltage at 1 cd m^−2^;

^b^External quantum efficiency, current efficiency and power efficiency: maximum, values at 1000 cd m^−2^;

^c^Commission Internationale de l’Eclairage 1931 coordinates at 1000 and 10,000 cd m^−2^;

^d^Correlated color temperature and color rendering index at 1000 cd m^−2^;

^e^Maximum luminance.

**Fig. 5:**
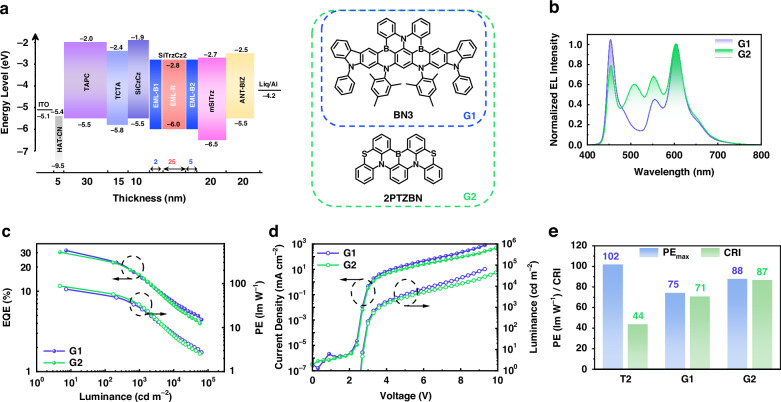
Device architecture and EL performance of WOLEDs incorporating deep blue MR emitter. **a** Device architecture, energy diagram and molecular structures of emitters. **b** EL spectra of devices G1 and G2 measured at a luminance of 1000 cd m^−2^. **c** EQE-luminance and PE-luminance (EQE-L-PE) curves of devices G1 and G2. **d** Current density-voltage and luminance-voltage (J-V-L) curves of devices G1 and G2. **e** Data comparison of PE_max_ and CRI values among devices T2, G1 and G2

Despite achieving outstanding EQE and PE in the devices described above, the high CRI and CIE coordinates meeting true-white-light criteria remain areas worth further exploration. To address this, we employed the same B-R-B EML structure strategy, adjusting the device configuration to incorporate the deep blue MR emitter BN3 (Fig. 5a) The BN3-based device displayed a deep blue emission at 458 nm with a FWHM of 23 nm^42^. In the white device G1, where the sky-blue emitter was replaced by 5 wt% BN3, a high EQE_max_ of 32.1% and PE_max_ of 74.5 lm W^−1^ were achieved with the CIE coordinate of (0.38, 0.30). The higher CRI of 71 was attributed to the spectral compensation from the deep blue light component (Fig. 5b). With the 0.5 wt% addition of a green MR-TADF dopant 2PTZBN into BN3-doped EML-B1 and -B2^43^, device G2 exhibited an impressive CRI of 88 and appropriate white light CIE coordinate of (0.37, 0.38). It was noteworthy that device G2 achieved a slightly lower EQE_max_ of 30.6% but a higher PE_max_ of 88.2 lm W^−1^ compared to G1, due to the green dopant reducing the required current density for the same brightness (Fig. 5c and d). In contrast to device T2, both devices achieved high levels of efficiency and CRI simultaneously (Fig. 5e). The stable emission spectra of both devices reconfirmed the success and reproducibility of the B-R-B structure (Fig. S12, Supporting Information). These results further validate our device design strategy for achieving highly efficient hybrid WOLEDs that deliver high-quality white light using MR-TADF emitters.

**Fig. 6:**
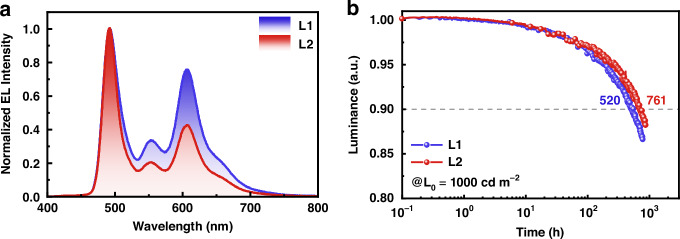
Device data and EL performance of device L1 and L2. **a** EL spectra of devices L1 and L2 at a luminance of 1000 cd m^−2^. **b** Operational lifetimes of devices L1 and L2 at the initial luminance of 1000 cd m^−2^

Finally, the operational lifetimes of hybrid WOLEDs based on the proposed B-R-B EML structure were evaluated. Here, an exciplex-host comprising DMIC-TRZ and DMIC-Cz was adopted to provide more balanced charge transport. The device structure and detailed data were shown in Fig. S11 (Supporting Information). Device L1 and L2 corresponded to BCzBN and BCzBN-3B, respectively. As a result, excellent EQEs and suppressed roll-offs were obtained. Device L1 and L2 achieved warm white light emission with CIE coordinates of (0.42, 0.44) and (0.36, 0.43), CRI values of 64 and 51, and CCT of 3415 K and 4715 K at the luminance of 1000 cd m^−2^, respectively (Fig. 6a). Delightfully, both devices secured exceptional operation lifetimes and maintained almost unchanged emission spectra after aging, depicted in Fig. S11c (Supporting Information). At an initial luminance of 1000 cd m^−2^, the LT_90_ values were 520 h for device L1 and 761 h for device L2 (Fig. 6b), which were significantly longer compared to those of the reported WOLEDs based on blue iridium complexes, and comparable to the most advanced hybrid or all-TADF WOLEDs (Table S3, Supporting Information). To the best of our knowledge, device L2 represents one of the best ultra-long-lifetime devices at this stage. The longer operational lifetime of device L2 compared to device L1 can be attributed to the superior properties of its blue light-emitting material, BCzBN-3B. The faster *k*_RISC_ of BCzBN-3B facilitates rapid consumption of triplet excitons, reducing their density and mitigating annihilation processes that degrade device performance.

## Discussion

In summary, we have successfully demonstrated color-stable hybrid WOLEDs with high PE and long device lifetime by using blue MR-TADF emitters and manipulating the exciton recombination zone. The devices based on BCzBN-3B showed stable white light emission over a wide range of 100–100,000 cd m^−2^, concurrently obtaining EQE_max/1000_ of 34.4%/23.4%, PE_max/1000_ of 101.8/44.8 lm W^−1^, and CIE coordinates of (0.31, 0.46). Owing to efficient energy transfer in the optimized device and the excellent structural stability of BCzBN-3B, the LT_90_ for BCzBN-3B is as long as 761 h. Furthermore, we achieved a high CRI of 88 with a CIE of (0.37, 0.38) and an EQE_max_ of 30.6%, demonstrating the potential of our strategy for producing high-quality white-light OLEDs. Our results underscore the significance of robust blue light emitters with short *τ*_d_ in building hybrid WOLEDs that possess high power efficiencies and extended operational lifetimes, paving the way for future applications in lighting and display.

## Materials and methods

### Materials and characterization

The materials used for fabricating the devices were either purchased from the company or synthesized by co-authors. The UV-vis absorption spectra were obtained on a Shimadzu UV-2600 spectrophotometer (Shimadzu, Japan) at room temperature with a concentration of 1 × 10^−5^ M. Phosphorescence spectra were measured on a Hitachi F-7100 fluorescence spectrophotometer at 77 K. The transient photoluminescence (PL) decay curves were obtained by FluoTime 300 (PicoQuant GmbH) with a Picosecond Pulsed UV-LASTER (LASTER375) as the excitation source. The solid-state *Φ*_PL_s were measured on a Hamamatsu UV-NIR absolute PL quantum yield spectrometer (C13534, Hamamatsu Photonics) equipped with an integrating sphere. The integrating sphere was purged with dry argon to maintain an inert atmosphere and all the samples were excited at 320 nm.

### Device fabrication and characterization

The ITO-coated glass substrates displayed a sheet resistance of 15 Ω square^−1^. Prior to deposition, these substrates underwent a thorough cleaning process, involving multiple cycles of ultrasonication with acetone and ethanol, followed by drying with nitrogen gas. The deposition process was operated at a pressure of 5 × 10^−5^ Pa. After the organic film was deposited at rates between 0.2 and 3 Å s^−1^, the Liq and Al layers were deposited at rates of 0.1 and 3 Å s^−1^, respectively. In this study, the emission area of each device was approximately 0.09 cm^2^. Measurements for current density-voltage-luminance (J-V-L), EQE/PE-L curves, and electroluminescence spectra were conducted using a Keithley 2400 source meter and an absolute EQE measurement system (C9920-12, Hamamatsu Photonics, Japan). The stability of the encapsulated WOLEDs was evaluated with an OLED lifetime test system (FS-MP64, Suzhou FSTAR Scientific Instrument Co. Ltd., China) under constant current density conditions, with an initial luminance of 1000 cd m^−2^.

## Supplementary information

The supplementary information

## Data Availability

The data that support the findings of this study are available from the corresponding author upon reasonable request.
