# Supplementary material for: High-power-efficiency and ultra-long-lifetime white OLEDs empowered by robust blue multi-resonance TADF emitters
Source: Light Sci Appl. 2025 Feb 11;14:81. doi: 10.1038/s41377-025-01750-z (PMC11814311; doi:10.1038/s41377-025-01750-z)
Supplement: Supplementary file 1 — The supplementary information [file 41377_2025_1750_MOESM1_ESM.docx]

**Supplementary Information for**

High-Power-Efficiency and Ultra-Long-Lifetime White OLEDs Empowered by Robust Blue Multi-Resonance TADF Emitters

Guohao Chen, Jingsheng Miao*, Xingyu Huang, Zhenghao Zhang, Zhuixing Xue, Manli Huang, Nengquan Li, Xiaosong Cao, Yang Zou and Chuluo Yang*

Shenzhen Key Laboratory of New Information Display and Storage Materials, College of Materials Science and Engineering, Shenzhen University, Shenzhen, 518060, P. R. China

Corresponding Authors: jingshengmiao@szu.edu.cn (Jingsheng Miao); clyang@szu.edu.cn (Chuluo Yang)

**Contents**

Analysis of rate constants

Fig. S1 Molecules used in the standard device structure.

Fig. S2 EL performance of blue device based on BCzCN.

Fig. S3 Device architecture diagram of lifetime-test devices and structures of molecules used.

Fig. S4 EL spectra of the device contained EML-R and -B with different thicknesses.

Fig. S5 EL spectra and performance of tri-color device replacing BCzBN with BCzBN-3B.

Fig. S6 EL spectra and performance of the tri-color device with different EML.

Fig. S7 EL performance of stable tri-color devices with different doping concentrations of BCzBN.

Fig. S8 EL performance of blue device based on BCzCN and BCzCN-3B.

Fig. S9 EL performance of stable tri-color devices with different doping concentrations of BCzBN-3B.

Fig. S10 EL spectra and performance of tri-color device T2 and W1-3.

Fig. S11 EL spectra and performance of lifetime-test devices with B-R-B structure.

Fig. S12 EL spectra of devices G1 and G2.

Fig. S13 Transient PL decay curves of doped films in the DMIC-TRZ matrix.

Fig. S14 Histograms displaying EQE variation in a batch of 9 devices with the B/R/B architecture for two emitters.

Table S1 Device configurations.

Table S2 Photophysical properties of BCzBN and BCzBN-3B.

Table S3 Summary of EL data for previously reported high-performance WOLEDs.

Table S4 Photophysical properties of doped films.

**Analysis of rate constants**

The rate constants of radiative decay (*k*_r,S_) and nonradiative decay (*k*_nr,S_) from S_1_ to S_0_ states, the rate constants of intersystem crossing (*k*_ISC_) and reverse intersystem crossing (*k*_RISC_) were calculated from the following six equations^1, 2^:

$k_{p}=1/\tau$_p_…………………………………………………………………Eq.(1)

$k_{d}=1/\tau$*_d_*…………………………………………………………………Eq.(2)

$k_{r,S}= \Phi_{p}k_{p}+\Phi_{d}k_{d}\approx\Phi_{p}k_{p}$…………………………… ………………Eq.(3)

$k_{nr,S}=\frac{1-\Phi_{\mathrm{PL}}}{\Phi_{\mathrm{PL}}}k_{r,S}$…………………………………………………………Eq.(4)

$k_{\mathrm{ISC}}=k_{p}-k_{r,S}-k_{nr,S}$……………………………….…………………Eq.(5)

$k_{\mathrm{RISC}}= \left( k_{p}k_{d}\Phi_{d} \right)/\left( k_{\mathrm{ISC}}\Phi_{p} \right)$……………………………………………Eq.(6)

Where *τ*_p_ and *τ*_d_ represent the prompt and decay fluorescence lifetime, which determined from transient PL spectra. The *k*_p_ and *k*_d_ represent the decay rate constants for prompt and delayed fluorescence, which could be calculated by Eq. (1) and (2). As can be seen from the formula, the longer *τ*_d_ is, the smaller *k*_d_ is obtained. *Φ*_p_ and *Φ*_d_ indicate prompt and delayed fluorescence components and can be distinguished from the total *Φ*_PL_ by comparing the integrated intensities of prompt and delayed components in the transient PL spectra. Therefore, *k*_r,S_, *k*_nr,S_, *k*_ISC_, *k*_RISC_ could be calculated by Eq.(3), (4) , (5) and (6) sequentially. The smaller *k*_d_ leads to a decreasing *k*_RISC_, representing that the shorter *τ*_d_ is vital to obtain a fast *k*_RISC_.

And *k*_FET_ can be calculated as follow:

$k_{\mathrm{FET}}=\frac{1}{\tau_{p,1}}-\frac{1}{\tau_{p,0}}$…………………………………………………………Eq.(7)

τ_p,1_ and τ_p,0_ represent prompt fluorescence decay times with or without guests, respectively.


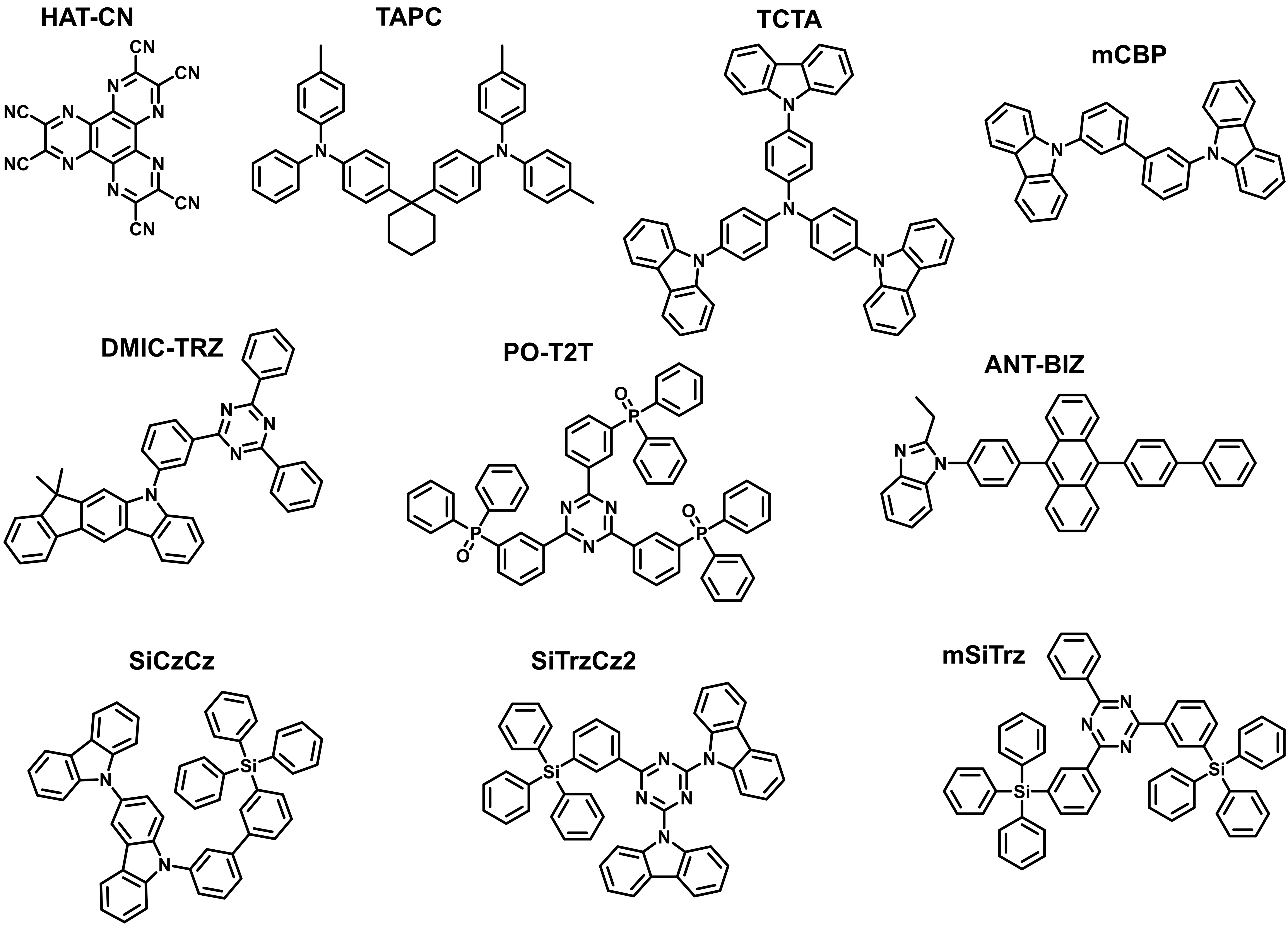


**Fig. S1 Molecules used in the standard device structure.**


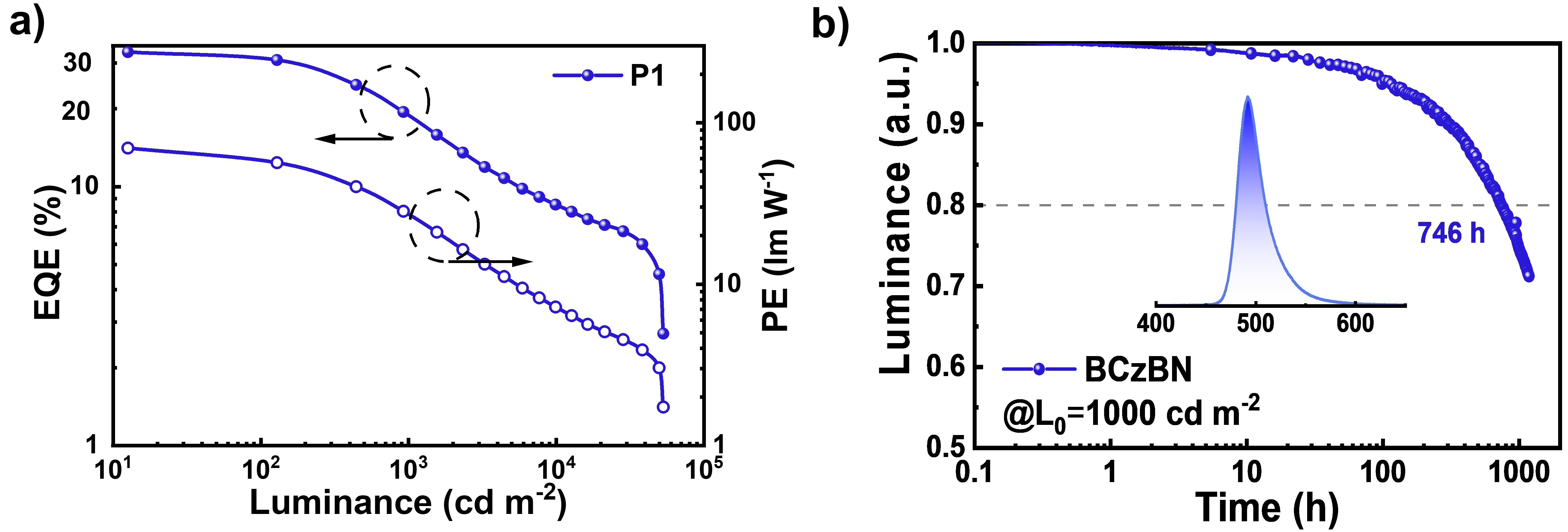


**Fig. S2 EL performance of blue device based on BCzCN. a** EQE-luminance and PE-luminance curves of device P1. **b** Operational lifetimes of BCzBN-based device. The insert shows EL spectra measured at a luminance of 1000 cd m^‒2^.

**
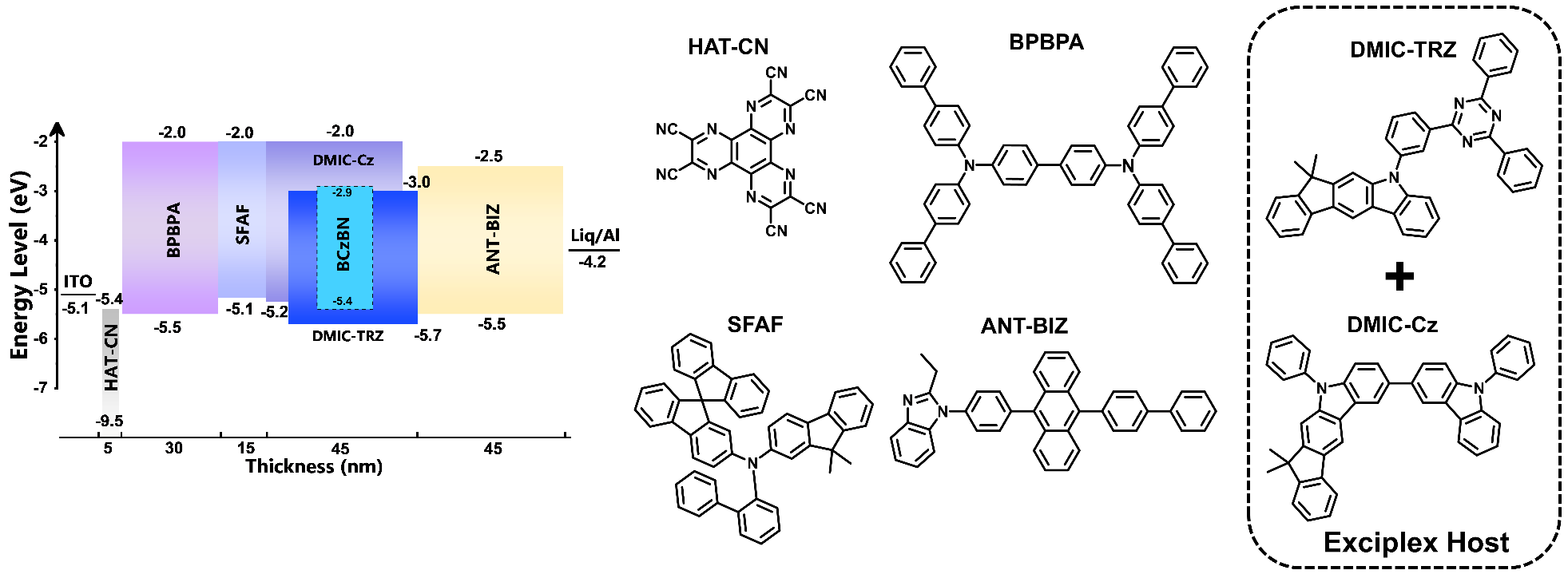
**

**Fig. S3** **Device architecture diagram of lifetime-test devices and structures of molecules used.**


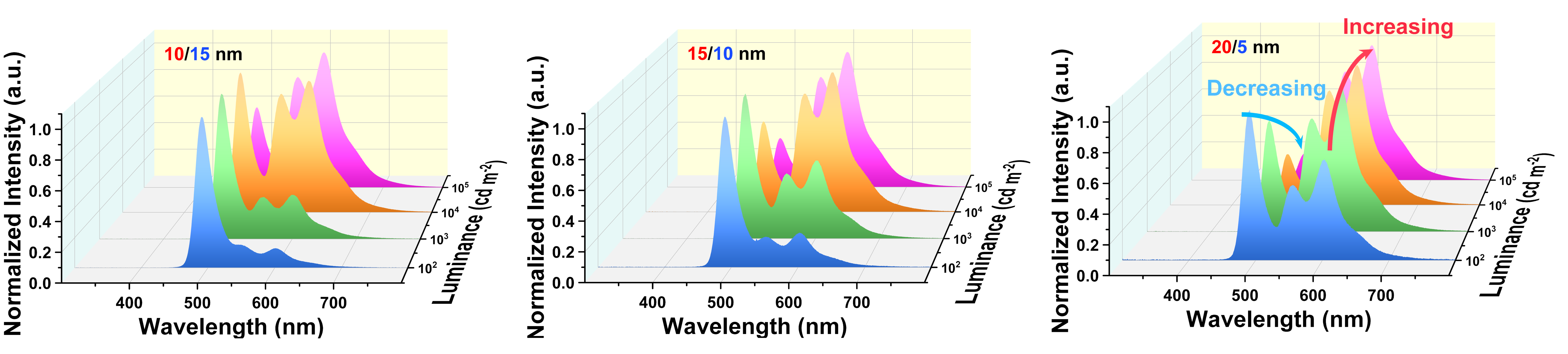


**Fig. S4 EL spectra of the device contained EML-R and -B with different thicknesses.** All devices showed decreasing blue emission and increasing red emission as the brightness increased.


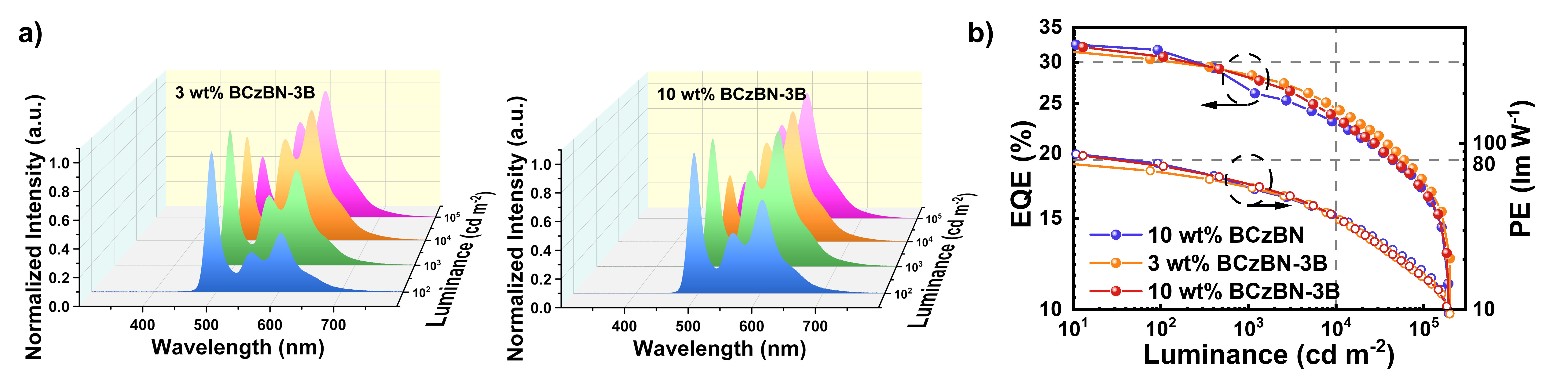


**Fig. S5 EL spectra and performance of tri-color device replacing BCzBN with BCzBN-3B. a** EL spectra of tri-color device replacing BCZBN with 3/10 wt% BCzBN-3B measured at a luminance of 10^2^, 10^3^, 10^4^ and 10^5^ cd m^‒2^. **b** EQE-luminance and PE-luminance (EQE-L-PE) curves of tri-color devices with 3/10 wt% BCzBN-3B compared the device with 10 wt% BCzBN.


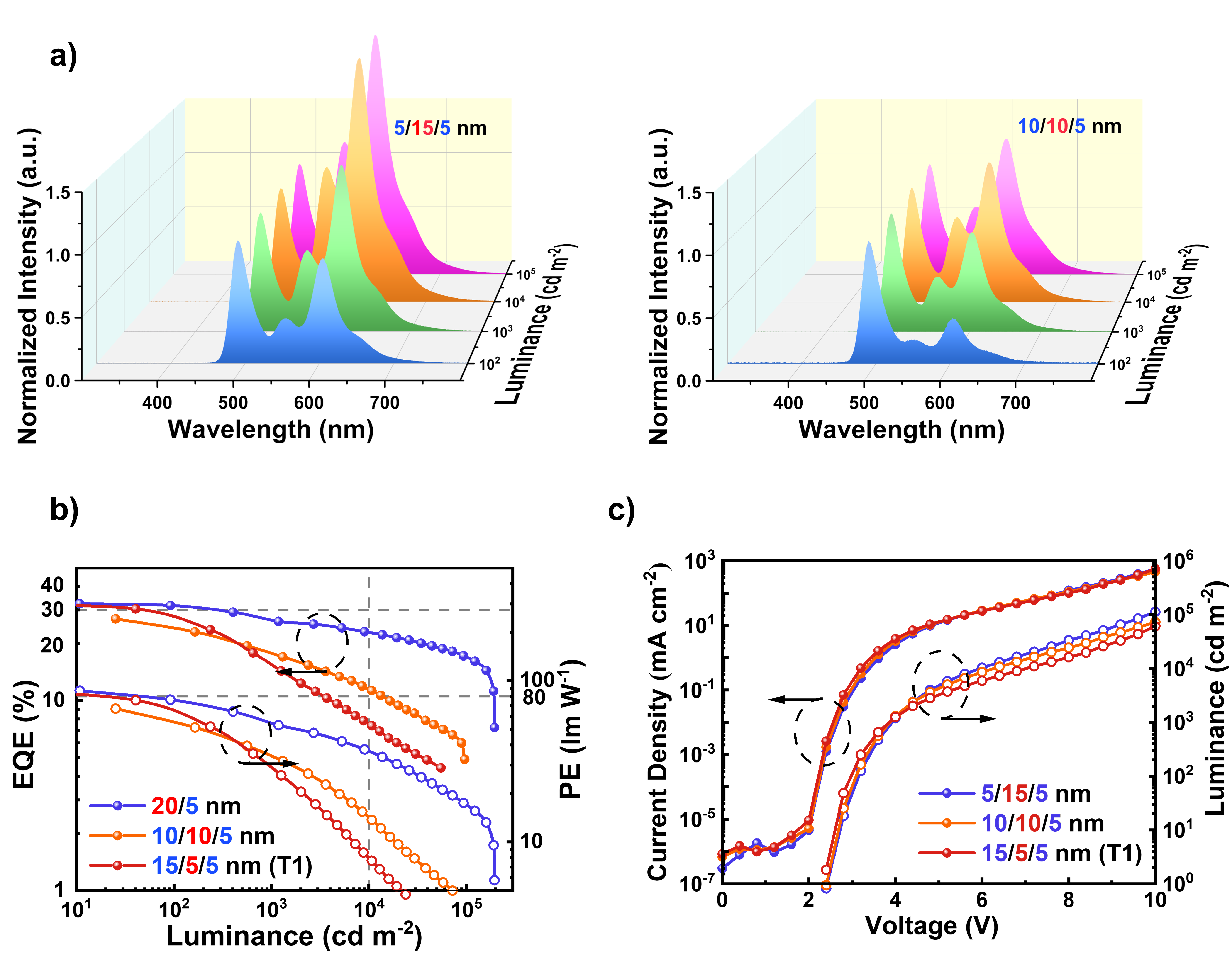


**Fig. S6 EL spectra and performance of the tri-color device with different EML-B1/R/B2.** **a** EL spectra of devices containing EML-B1/R/B2 with different thicknesses measured at a luminance of 10^2^, 10^3^, 10^4^ and 10^5^ cd m^‒2^. **b** EQE-luminance and PE-luminance (EQE-L-PE) curves of devices containing EML-B1/R/B2 with different thicknesses. **c** Current density-voltage and luminance-voltage (J-V-L) curves of devices containing EML-B1/R/B2 with different thicknesses.


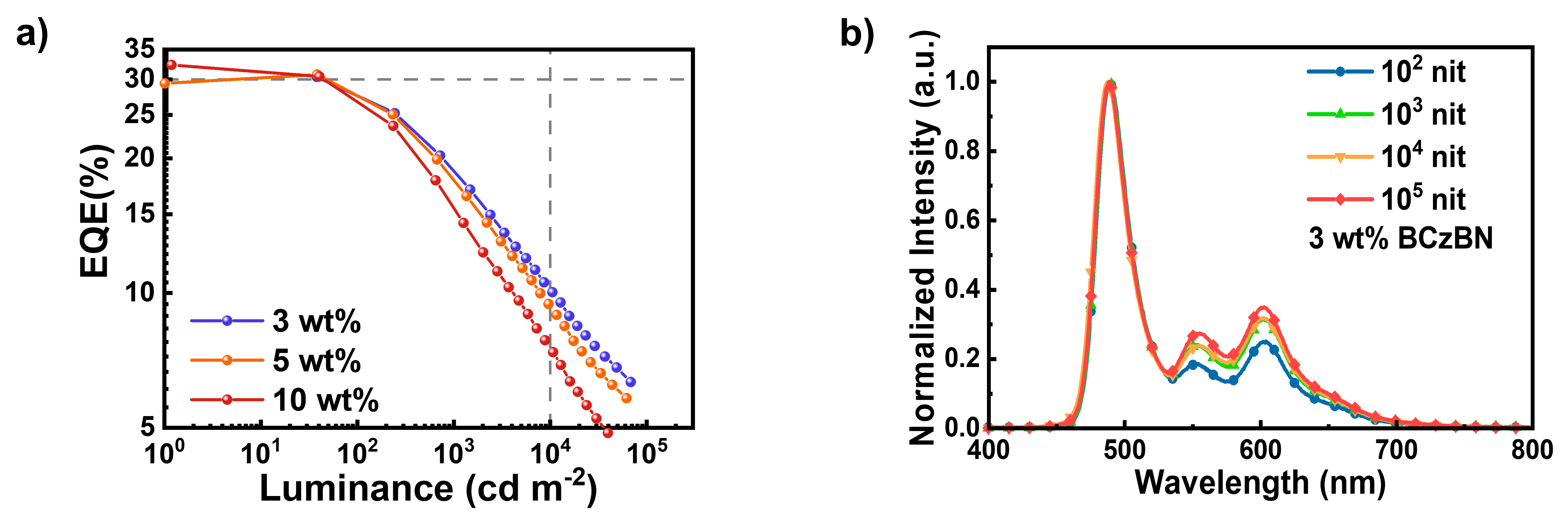


**Fig. S7 EL performance of stable tri-color devices with different doping concentrations of BCzBN.** **a** EQE-luminance (EQE-L) curves of stable tri-color devices with 3/5/10 wt% doping concentration of BCzBN. **b** EL spectra of the device with 3 wt% BCzBN measured at a luminance of 10^2^, 10^3^, 10^4^ and 10^5^ cd m^‒2^.


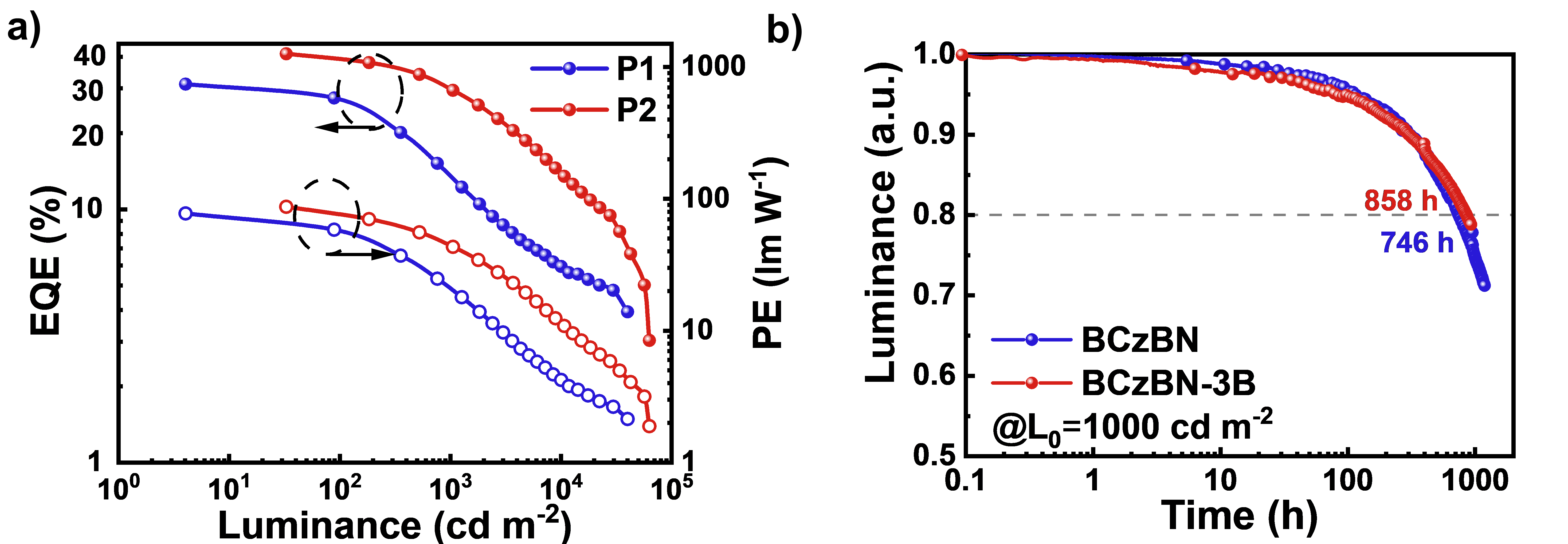


**Fig. S8 EL performance of blue device based on BCzCN and BCzCN-3B. a** EQE-luminance and PE-luminance (EQE-L-PE) curves of devices P1 and P2. **b** Operational lifetimes of lifetime-test devices based on BCzBN and BCzBN-3B.


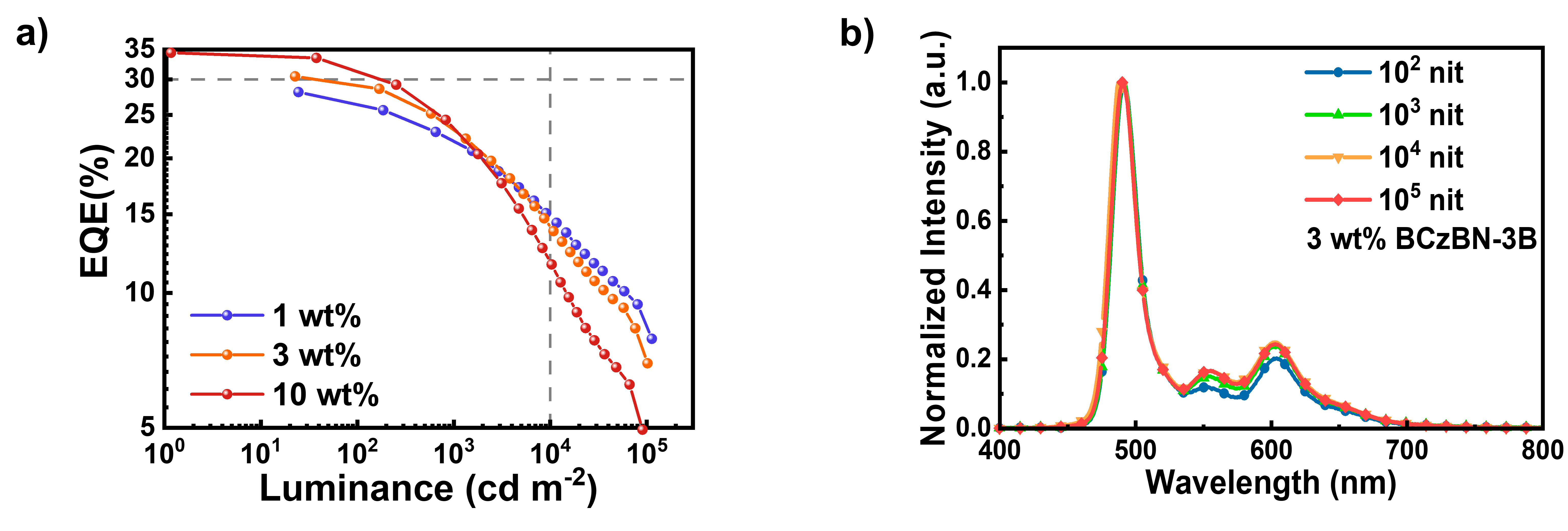


**Fig. S9 EL performance of stable tri-color devices with different doping concentrations of BCzBN-3B.** **a** EQE-luminance (EQE-L) curves of stable tri-color devices with 1/3/10 wt% doping concentration of BCzBN-3B. **b** EL spectra of the device with 3 wt% BCzBN-3B measured at a luminance of 10^2^, 10^3^, 10^4^ and 10^5^ cd m^‒2^.


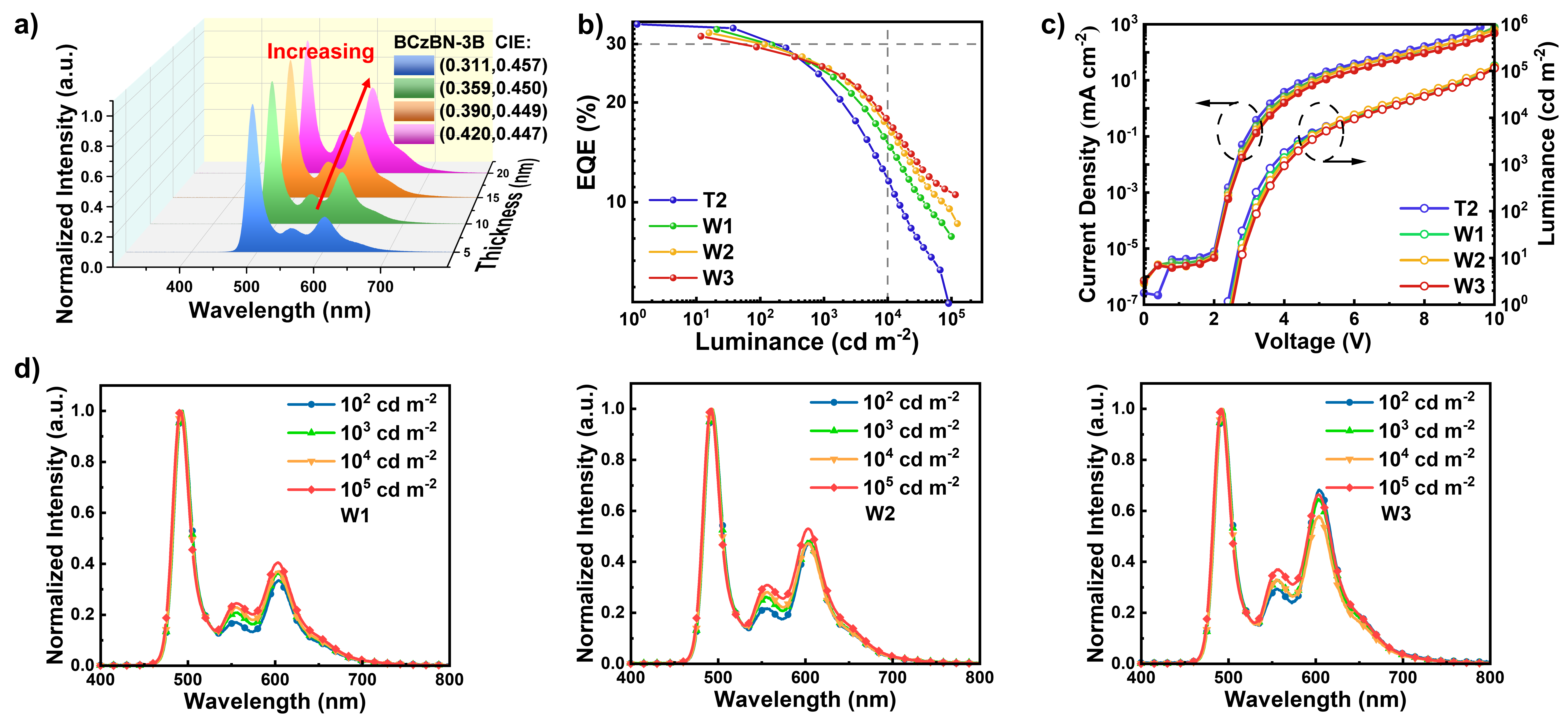


**Fig. S10 EL spectra and performance of tri-color device T2 and W1-3. a** EL spectra of the device based on T2 with different thicknesses of EML-R measured at a luminance of 1000 cd m^‒2^. The relative percentage of red emission increased with thickness. **b** EQE-luminance (EQE-L) curves of devices T2 (5 nm), W1 (10 nm), W2 (15 nm) and W3 (20 nm) with different thicknesses of EML-R. **c** Current density-voltage and luminance-voltage (J-V-L) curves of device T2, W1, W2 and W3. **d** EL spectra of devices W1, W2 and W3 measured at a luminance of 10^2^, 10^3^, 10^4^ and 10^5^ cd m^‒2^.


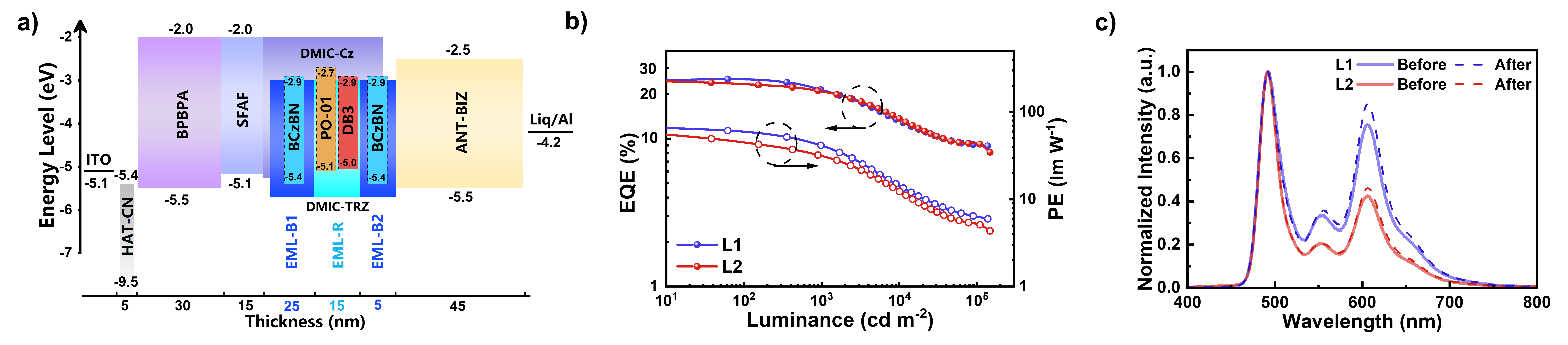


**Fig. S11 EL spectra and performance of lifetime-test devices with B-R-B structure.** **a** Device architecture. **b** EQE-luminance and PE-luminance (EQE-L-PE) curves of devices L1 and L2. **c** EL spectra for devices L1 and L2 before and after aging.


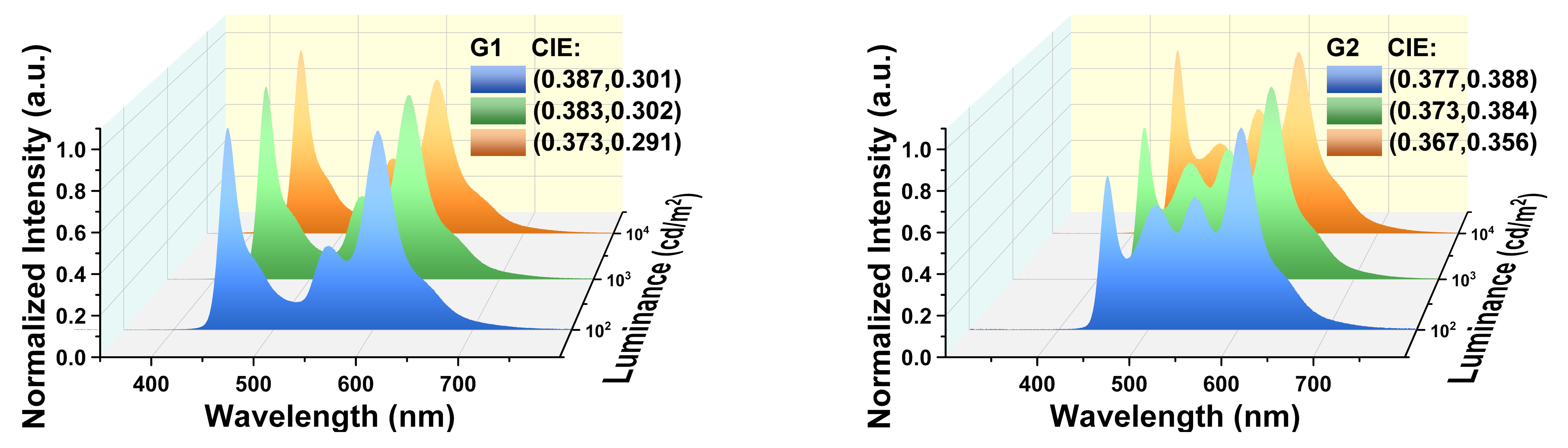


**Fig. S12 EL spectra of devices G1 and G2.** EL spectra of devices G1 and G2 measured at a luminance of 10^2^, 10^3^ and 10^4^ cd m^‒2^.


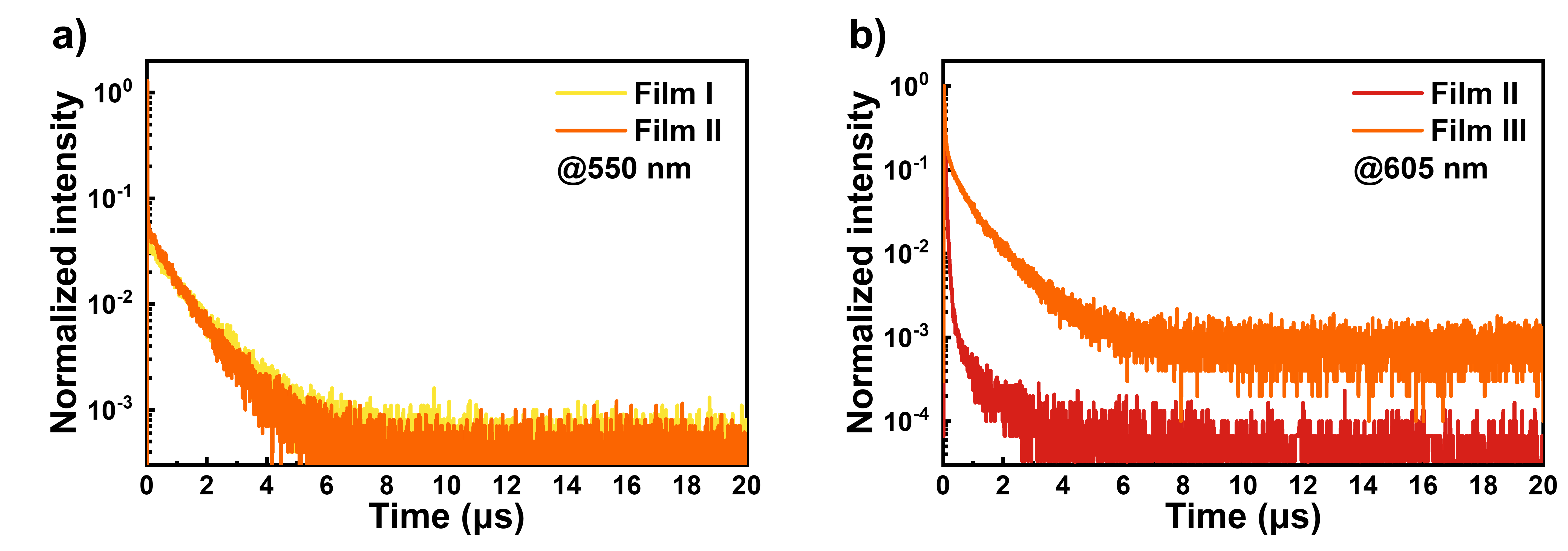


**Fig. S13 Transient PL decay curves of doped films in the DMIC-TRZ matrix.** **a** Lifetimes of prompt fluorescent at the emission wavelength of 550 nm for Film I (1 wt% PO-01: DMIC-TRZ) and Film II (0.3 wt% DB3: 1 wt% PO-01: DMIC-TRZ). **b** Lifetimes of prompt and delayed fluorescent at the emission wavelength of 605 nm for Film II and Film III (0.3 wt% DB3: DMIC-TRZ).


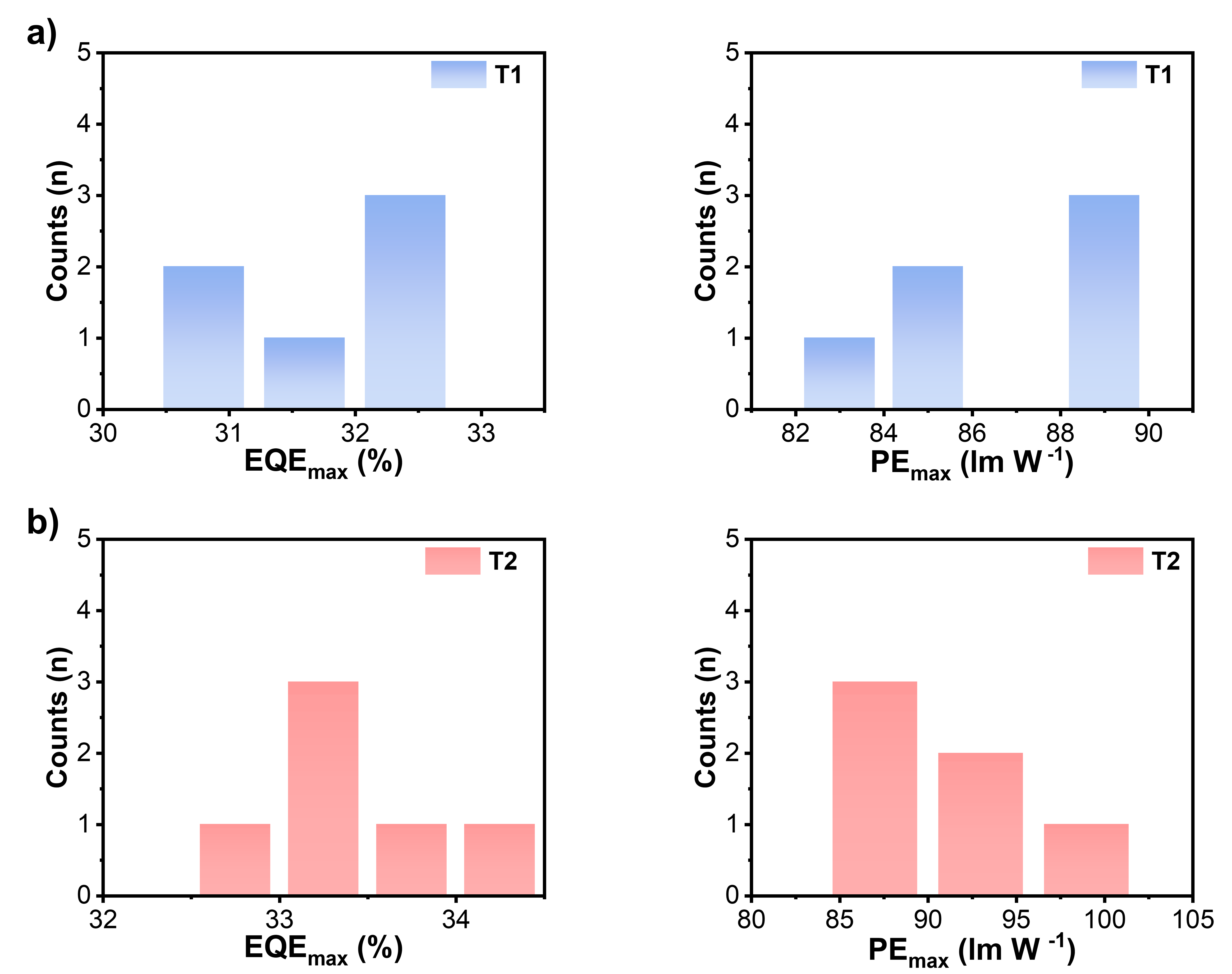


**Fig. S14 Histograms displaying EQE and PE variation in a batch of 6 devices with the B/R/B architecture for two emitters. a** Statistical data on EQE_max_ and PE_max_ for BCzBN (T1); **b** Statistical data on EQE_max_ and PE_max_ for BCzBN-3B (T2).

**Table S1 Device configurations.**

| P1 | ITO/HAN-CN (5 nm)/TAPC (30 nm)/TCTA (15 nm)/mCBP (10 nm)/2 wt% BCzBN: DMIC-TRZ (45 nm)/ POT2T (20 nm)/ANT-BIZ (30 nm)/Liq (2 nm)/Al |
| --- | --- |
| P2 | ITO/HAN-CN (5 nm)/TAPC (30 nm)/TCTA (15 nm)/mCBP (10 nm)/2 wt% BCzBN-3B: DMIC-TRZ (45 nm)/ POT2T (20 nm)/ANT-BIZ (30 nm)/Liq (2 nm)/Al |
| D1 | ITO/HAN-CN (5 nm)/TAPC (30 nm)/TCTA (15 nm)/mCBP (10 nm)/0.3 wt% P0-01: 5 wt% BCZBN: DMIC-TRZ (25 nm)/POT2T (20 nm)/ANT-BIZ (30 nm)/Liq (2 nm)/Al |
| D2 | ITO/HAN-CN (5 nm)/TAPC (30 nm)/TCTA (15 nm)/mCBP (10 nm)/0.5 wt% P0-01: 5 wt% BCZBN: DMIC-TRZ (25 nm)/POT2T (20 nm)/ANT-BIZ (30 nm)/Liq (2 nm)/Al |
| T1 | ITO/HAN-CN (5 nm)/TAPC (30 nm)/TCTA (15 nm)/mCBP (10 nm)/10 wt% BCzBN: DMIC-TRZ (15 nm)/0.3 wt% DB3: 1 wt% PO-01: DMIC-Trz (5 nm)/10 wt% BCzBN: DMIC-TRZ (5 nm)/POT2T (20 nm)/ANT-BIZ (30 nm)/Liq (2 nm)/Al |
| T2 | ITO/HAN-CN (5 nm)/TAPC (30 nm)/TCTA (15 nm)/mCBP (10 nm)/10 wt% BCzBN-3B: DMIC-TRZ (15 nm)/0.3 wt% DB3:1 wt% PO-01: DMIC-Trz (5 nm)/10 wt% BCzBN-3B: DMIC-TRZ (5 nm)/POT2T (20 nm)/ANT-BIZ (30 nm)/Liq (2 nm)/Al |
| W1 | ITO/HAN-CN (5 nm)/TAPC (30 nm)/TCTA (15 nm)/mCBP (10 nm)/10 wt% BCzBN-3B: DMIC-TRZ (15 nm)/0.3 wt% DB3: 1 wt% PO-01: DMIC-Trz (10 nm)/10 wt% BCzBN-3B: DMIC-TRZ (5 nm)/POT2T (20 nm)/ANT-BIZ (30 nm)/Liq (2 nm)/Al |
| W2 | ITO/HAN-CN (5 nm)/TAPC (30 nm)/TCTA (15 nm)/mCBP (10 nm)/10 wt% BCzBN-3B: DMIC-TRZ (15 nm)/0.3 wt% DB3: 1 wt% PO-01: DMIC-Trz (15 nm)/10 wt% BCzBN-3B: DMIC-TRZ (5 nm)/POT2T (20 nm)/ANT-BIZ (30 nm)/Liq (2 nm)/Al |
| W3 | ITO/HAN-CN (5 nm)/TAPC (30 nm)/TCTA (15 nm)/mCBP (10 nm)/10 wt% BCzBN-3B: DMIC-TRZ (15 nm)/0.3 wt% DB3: 1 wt% PO-01: DMIC-Trz (20 nm)/10 wt% BCzBN-3B: DMIC-TRZ (5 nm)/POT2T (20 nm)/ANT-BIZ (30 nm)/Liq (2 nm)/Al |
| G1 | ITO/HAN-CN (5 nm)/TAPC (30 nm)/TCTA (15 nm)/SiCzCz (15 nm)/5 wt% BN3: SiTrzC2 (2 nm)/0.3 wt% DB3: 1 wt% PO-01: SiTrzC2 (25 nm)/5 wt% BN3: SiTrzC2 (5 nm)/mSiTrz (20 nm)/ANT-BIZ (20 nm)/Liq (2 nm)/Al |
| G2 | ITO/HAN-CN (5 nm)/TAPC (30 nm)/TCTA (15 nm)/SiCzCz (15 nm)/0.5 wt% 2PTZBN: 5 wt% BN3: SiTrzC2 (2 nm)/0.3 wt% DB3: 1 wt% PO-01: SiTrzC2 (25 nm)/0.5 wt% 2PTZBN: 5 wt% BN3: SiTrzC2 (5 nm)/mSiTrz (20 nm)/ANT-BIZ (20 nm)/Liq (2 nm)/Al |
| L1 | ITO/HAN-CN (5 nm)/BPBPA (30 nm)/SFAF (15 nm)/5 wt% BCzBN: DMIC-TRZ (25 nm)/0.3 wt% DB3:1 wt% PO-01: DMIC-Trz (15 nm)/5 wt% BCzBN: DMIC-TRZ (5 nm)/ANT-BIZ (45 nm)/Liq (2 nm)/Al |
| L2 | ITO/HAN-CN (5 nm)/BPBPA (30 nm)/SFAF (15 nm)/5 wt% BCzBN-3B: DMIC-TRZ (25 nm)/0.3 wt% DB3:1 wt% PO-01: DMIC-Trz (15 nm)/5 wt% BCzBN-3B: DMIC-TRZ (5 nm)/ANT-BIZ (45 nm)/Liq (2 nm)/Al |

**Table S2 Photophysical properties of BCzBN and BCzBN-3B.**

| Emitter | 𝜆_abs_ ^[a]^  [nm] | 𝜆_em_ ^[a]^  [nm] | FWHM ^[a]^  [nm]/[meV] | *∆E*_ST_ ^[a]^  [eV] | *Φ*_PL_ ^[b]^  [%] | *Θ*_//_ ^[b]^  [%] | *τ*_d_ ^[b]^  [μs] | *k*_r,S_ ^[b]^  [10^7^ s^‒1^] | | *k*_ISC_^[b]^  [10^7^ s^‒1^] | *k*_RISC_^[b]^  [10^4^ s^‒1^] | |
| --- | --- | --- | --- | --- | --- | --- | --- | --- | --- | --- | --- | --- |
| BCzBN | 467 | 484 | 23/0.12 | 0.14 | 91 | 88 | 76 | 2.42 | 10.8 | | 6.35 |  |
| BCzBN-3B | 470 | 482 | 16/0.09 | 0.10 | 95 | 94 | 40 | 2.30 | 7.02 | | 9.52 |  |

[a] Peak of absorption (*λ*_abs_), fluorescence (*λ*_em_, 300 K) spectra, full-width at half-maximum (FWHM) of fluorescence, and S_1_-T_1_ energy gap (*ΔE*_ST_) measured in 10^‒5^ mol L^‒1^ toluene solutions; [b] absolute photoluminescence quantum yield (*Φ*_PL_), horizontal orientation factor (*Θ*_//_), delayed fluorescence lifetime (*τ*_d_), rate constants of singlet radiative decay (*k*_r,S_), intersystem crossing (*k*_ISC_), reverse intersystem crossing (*k*_RISC_) measured in 2 wt% emitter: DMIC-TRZ films; note: the quantum yield were measured under deoxygenated condition.

**Table S3** **Summary of EL data for previously reported high-performance WOLEDs.**

| Ref. | | V_on_^[a]^  [V] | L_max_^[a]^  [cd m^‒2^] | EQE_max/1000_^[b]^  [%] | PE_max/1000_^[b]^  [lm W^‒1^] | CIE^[c]^ | Lifetime^[c]^  [h] | |
| --- | --- | --- | --- | --- | --- | --- | --- | --- |
| This work | BCzBN | 2.4 | 63,777 | 32.3/14.4 | 89.1/25.9 | (0.33, 0.45) | | 520 (LT_90_) |
|  | BCzBN-3B | 2.4 | 90,137 | 34.4/23.4 | 101.8/44.8 | (0.31, 0.46) | | 740 (LT_90_) |
| Hybrid | 3 | 2.6 | 35,200 | 25.4/25.2 | 71.0/49.7 | (0.51, 0.42) | | - |
|  | 4 | 2.4 | 9,501 | 24.5/24.3 | 64.0/49.2 | (0.54, 0.41) | | 17,687 (LT_95_) |
|  | 5 | 2.6 | - | 23.6/18.3 | 68.8/38.1 | (0.49, 0.43) | | 600 (LT_50_) |
|  | 6 | 2.6 | 37,782 | 23.6/20.0 | 77.8/49.5 | (0.47, 0.41) | | - |
|  | 7 | 2.6 | 20,000 | 23.4/20.3 | 50.4/31.2 | (0.49, 0.43) | | - |
|  | 8 | 3.1 | - | 20.3/20.1 | 40.3/- | (0.38, 0.45) | | - |
|  | 9 | 2.6 | 20,510 | 22.3/19.6 | 86.7/69.2 | (0.44, 0.48) | | - |
|  | 10 | 3.1 | 14,840 | 26.8/18.7 | 58.0/26.6 | (0.39, 0.39) | | - |
|  | 11 | 2.7 | - | 19.6/15.4 | 52.2/29.1 | (0.33, 0.45) | | 2,304 (LT_50_) |
| All TADF | 12 | 2.7 | 37,000 | 32.7/29.6 | 108.2/39.5 | (0.44, 0.47) | | 233 (LT_50_) |
|  | 13 | 2.5 | - | 21.7/21.4 | 69.6/41.5 | (0.42,0.44) | | 8,284 (LT_80_) |
|  |  | 2.5 |  | 16.7/16.5 | 49.8/39.3 | (0.36,0.43) | | 3,131 (LT_80_) |
|  | 14 | 2.6 | - | 32.8/24.1 | 99.9/- | (0.41, 0.46) | | - |
|  | 15 | 2.5 | - | 20.5/13.0 | 59.6/31.7 | (0.33, 0.41) | | 2,283 (LT_50_) |
|  | 16 | 2.7 | 57,830 | 31.0/18.7 | 77.5/32.8 | (0.43,0.41) | | 125.2 (LT_70_) |
|  |  | 2.8 | 54,950 | 25.5/16.0 | 46.1/24.9 | (0.47,0.42) | | 205.2 (LT_70_) |
|  | 17 | - | - | 30.7/30.3 | 57.7/51.2 | (0.31, 0.37) | | 402 (LT_80_) |
|  | 18 | 2.6 | 52,690 | 31.1/25.3 | 130.7/78.5 | (0.37, 0.51) | | 1,548 (LT_50_) |
|  | 19 | 2.8 | 49,449 | 30.3/15.1 | 64.2/- | (0.35, 0.44) | | 8.8 (LT_80_) |
| All Phos. | 20 | 2.5 | - | 28.1/21.5 | 105.0/59.5 | (0.40, 0.48) | | - |
|  | 21 | 2.7 | 25,540 | 25.6/25.1 | 48.3/24.1 | (0.41, 0.46) | | - |

[a] turn-on voltages and maximum luminance; [b] external quantum efficiency and power efficiency at maximum value and 1000 cd m^‒2^. [c] commission Internationale de l’Eclairage coordinates and operational lifetime at 1000 cd m^‒2^.

**Table S4 Photophysical properties of doped film.**

| Film | | *Φ*_PL_^[a]^  [%] | *τ*_p_^[a]^  [ns] | *k*_r_^[a]^  [s^‒1^] | *k*_nr_^[a]^  [s^‒1^] | *k*_FRET_^[b]^  [s^‒1^] |
| --- | --- | --- | --- | --- | --- | --- |
| I | 1 wt% PO-01: DMIC-TRZ | 87 | 1,158 | 7.51 × 10^5^ | 1.12 × 10^5^ | 1.49 × 10^5^ |
| II | 0.3 wt% DB3:1 wt%PO-01: DMIC-TRZ | 89 | 988 | 9.01 × 10^5^ | 1.01 × 10^5^ |  |

[a] absolute photoluminescence quantum yield (*Φ*_PL_), prompt fluorescence lifetime (*τ*_p_), rate constants of radiative decay (*k*_r_) and nonradiative decay (*k*_nr_); [b] rate constants of Förster-type energy transfer.

**References**

1. Tsuchiya Y*, et al.* Exact solution of kinetic analysis for thermally activated delayed fluorescence materials. *J Phys Chem A* **125**, 8074-8089 (2021).

2. Aizawa N, Shikita S, Yasuda T. Spin-dependent exciton funneling to a dendritic fluorophore mediated by a thermally activated delayed fluorescence material as an exciton-harvesting host. *Chem Mater* **29**, 7014-7022 (2017).

3. Zhang H*, et al.* Novel Deep-blue hybridized local and charge-transfer host emitter for high-quality fluorescence/phosphor hybrid quasi-white organic light-emitting diode. *Adv Funct Mater* **31**, 2100704 (2021).

4. Ameri L, Cao L, Tan X, Li J. Efficient, color-stable, and long-lived white organic light-emitting diodes utilizing phosphorescent molecular aggregates. *Adv Mater* **35**, 2208361 (2023).

5. Chen Y, Yang D, Qiao X, Dai Y, Sun Q, Ma D. Novel strategy to improve the efficiency roll-off at high luminance and operational lifetime of hybrid white OLEDs via employing an assistant layer with triplet–triplet annihilation up-conversion characteristics. *J Mater Chem C* **8**, 6577-6586 (2020).

6. Chen Y*, et al.* Highly efficient fluorescence/phosphorescence hybrid white organic light-emitting devices based on a bipolar blue emitter to precisely control charges and excitons. *J Mater Chem C* **8**, 7543-7551 (2020).

7. Chen Y, Sun Q, Dai Y, Yang D, Qiao X, Ma D. EL properties and exciton dynamics of high-performance doping-free hybrid woleds based on 4P-NPD/Bepp2 heterojunction as blue emitter. *Adv Opt Mater* **7**, 1900703 (2019).

8. Wang Q*, et al.* Alleviating efficiency roll-off of hybrid single-emitting layer woled utilizing bipolar tadf material as host and emitter. *ACS Appl Mater Interfaces* **11**, 2197-2204 (2019).

9. Xu Z*, et al.* High efficiency and low roll-off hybrid woleds by using a deep blue aggregation-induced emission material simultaneously as blue emitter and phosphor host. *Adv Opt Mater* **7**, 1801539 (2019).

10. Liu Z*, et al.* Simultaneously realizing high efficiency and high color rendering index for hybrid white organic light-emitting diodes by ultra-thin design of delayed fluorescence sensitized phosphorescent layers. *Small* **20**, 2305589 (2024).

11. Wei P, Zhang D, Duan L. Modulation of Förster and Dexter interactions in single-emissive-layer all-fluorescent woleds for improved efficiency and extended lifetime. *Adv Funct Mater* **30**, 1907083 (2020).

12. Han C*, et al.* Ladder-like energy-relaying exciplex enables 100% internal quantum efficiency of white TADF-based diodes in a single emissive layer. *Nat Commun* **12**, 3640 (2021).

13. Zhang C*, et al.* A π–D and π–A exciplex-forming host for high-efficiency and long-lifetime single-emissive-layer fluorescent white organic light-emitting diodes. *Adv Mater* **32**, 2004040 (2020).

14. Chen J*, et al.* Thermally activated delayed fluorescence warm white organic light emitting devices with external quantum efficiencies over 30%. *Adv Funct Mater* **31**, 2101647 (2021).

15. Wu Z*, et al.* Strategic-tuning of radiative excitons for efficient and stable fluorescent white organic light-emitting diodes. *Nat Commun* **10**, 2380 (2019).

16. Liu H, Fu Y, Tang BZ, Zhao Z. Realizing high efficiency and high color quality for all-fluorescence white organic light-emitting diodes by interlayer-sensitizing configuration with electron-capturing agent. *Adv Funct Mater* **33**, 2309770 (2023).

17. Zhang C*, et al.* Color-tunable all-fluorescent white organic light-emitting diodes with a high external quantum efficiency over 30% and extended device lifetime. *Adv Mater* **34**, 2103102 (2022).

18. Liu H, Fu Y, Tang BZ, Zhao Z. All-fluorescence white organic light-emitting diodes with record-beating power efficiencies over 130 lm W^‒1^ and small roll-offs. *Nat Commun* **13**, 5154 (2022).

19. Liu D*, et al.* Highly horizontal oriented tricomponent exciplex host with multiple reverse intersystem crossing channels for high-performance narrowband electroluminescence and eye-protection white organic light-emitting diodes. *Adv Mater*, 2403584 (2024).

20. Wu S-F*, et al.* White organic LED with a luminous efficacy exceeding 100 lm W^−1^ without light out-coupling enhancement techniques. *Adv Funct Mater* **27**, 1701314 (2017).

21. Liang J, Li C, Zhuang X, Ye K, Liu Y, Wang Y. Novel blue bipolar thermally activated delayed fluorescence material as host emitter for high-efficiency hybrid warm-white OLEDs with stable high color-rendering index. *Adv Funct Mater* **28**, 1707002 (2018).
